# The Prevention of Noise Induced Hearing Loss in Children

**DOI:** 10.1155/2012/473541

**Published:** 2012-12-13

**Authors:** Robert V. Harrison

**Affiliations:** Auditory Science Laboratory, Department of Otolaryngology-Head & Neck Surgery, University of Toronto and The Hospital for Sick Children, 555 University Avenue, Toronto, ON, Canada M5G 1X8

## Abstract

Increasingly, our acoustic environment is filled with amplified sound sources (e.g., MP3 players, video game stations, and sports/entertainment venues). There is serious concern and also some controversy about the risks of acoustic trauma in children. This overview provides some basic information on the physiological mechanisms that lead to noise induced hearing loss, a survey of various studies that, on balance, indicates that there is cause for concern, and finally a discussion on measures that can help to prevent noise induced hearing loss in children. This paper is designed for public health and other healthcare professions (ENT, audiologists, family doctors, and pediatricians) who should understand the risks of noise induced hearing loss and its prevention.

## 1. Introduction

In most of our societies (both developed and developing) there are increasing levels of acoustic signal exposure, in large part due to the (electronic) amplification of sound and to the ubiquitous use of audio entertainment devices. Attention has shifted from the noise exposure problems in specific groups, for example, in industrial or military environments, to a more widespread potential source of noise trauma. A variety of sound sources, for example, earphones and in-door and outdoor loudspeakers, are capable of decibel levels that can result in acoustic trauma if not used carefully. This paper is provided for public health and hearing healthcare professionals so that they can more fully understand the problem of noise induced hearing loss and provide authoritative advice. A range of the literature is reviewed including epidemiological studies, data from hearing related questionnaires and surveys, audiology case studies, and reports of (noise induced) tinnitus. This paper also provides advice on how to prevent noise induced hearing loss, both for patients/parents and in educational programs for school children. To start, here is an overview of physiological mechanisms involved in acoustic damage to the cochlea.

## 2. Acoustic Trauma to the Cochlea

Many of the earliest auditory science studies were carried out to explore the effects of acoustic trauma, for example, going back to work by Davis and colleagues in 1935 [2]. There is now very extensive anatomical and physiological data on the consequences of acoustic trauma. However, there appear to be no simple rules that relate the type or level of noise exposure with the degree of cochlear dysfunction or anatomical damage. What is clear is the traumatizing effect of an acoustic signal can be different depending on both the spectral and temporal aspects of the signal, as well as exposure duration. High intensity impulsive signals can cause direct mechanical damage to the cochlea, as can less intense noise signals over a long period of exposure. Relatively low levels of noise for long periods may not result in direct mechanical damage but rather induce metabolic changes in sensory cells that might eventually recover or, alternatively, initiate cell apoptosis. If the metabolic and mechanical effects of noise trauma could be separated, it might be possible to formulate some rules that generally describe the effects of acoustic trauma. However, metabolic and mechanical events in the cochlea are intimately linked, making such distinctions difficult. In short, the vast literature describing the anatomical and physiological effects of acoustic trauma cannot easily be distilled into a neat summary. The present overview draws on some key studies that give us important insights into the nature of acoustic trauma. Below is a review of some of the anatomical changes that can be seen in the noise traumatized cochlea as well as the functional deficits revealed in electrophysiological studies.

## 3. Anatomical Damage Caused by Acoustic Trauma

Very intense acoustic signals (>130 dB SPL) can directly cause mechanical damage to the cochlea as well as to middle-ear structures. The initial insult may not be directly to hair cells, but to other supporting structures in the organ of Corti as well as Reissner's and tectorial membranes. Hair cell and neuronal degeneration may follow subsequently, perhaps due to mixing of endolymph and perilymph or the release of cytotoxic agents (e.g., free radicals; excessive amounts of neurotransmitter) from damaged tissue. In the context of “recreational” noise exposure (MP3 players, etc.), it is unlikely that stimuli will reach levels to cause direct lesions to the organ of Corti. Less intense acoustic signals, especially if impulsive (high concentration of energy in the time domain) or with dominant spectral energy peaks (overstimulating local frequency-specific cochlear regions) can mechanically damage hair cells, with the most vulnerable structures being the stereocilia.

Milder acoustic exposure for a long duration can cause intracellular changes in hair cells related to, for example, metabolic depletion or excessive release of neurotransmitter. Up to a certain point, metabolic damage in hair cells is reversible, and as such may be manifested in a recovery from a temporary threshold shift. However at some tipping point, apoptosis (programmed cell death) will occur and lead to complete hair cell loss. The story does not end here. Any damage at the cochlear level leads to central auditory pathways alterations. Most locally, excess release of glutamate from overstimulated inner hair cells can cause excitotoxicity to the cochlear afferent neurons. With such deafferentation, degenerative changes can be observed in second- and third-order neurons throughout the auditory brainstem and midbrain. Some of these pathophysiological events are discussed in more detail below.

## 4. Changes within the Hair Cells

A number of ultrastructural changes to cochlear hair cells resulting from noise exposure can be attributed to metabolic exhaustion [3–5]. These include mitochondrial damage that can reflect changes in cell energy production and an increase in vacuoles of the endoplasmic reticulum that could indicate deficits in protein synthesis. Such changes can be found in outer hair cells after relatively short durations (1 min) exposure to broadband noise at levels of 130 dB SPL [6]. It is not clear whether these signs of metabolic exhaustion result from direct effects of hair cell overstimulation or are secondary to a more local hypoxia/ischemia. Some evidence for the latter is that cochlear afferent dendrites near the inner hair cells often show swellings similar to changes produced by general cochlear hypoxia. In any event, there is breakdown in the cytoplasmic processes that maintain cellular integrity. Long durations of noise exposure (e.g., one hour at 130 dB SPL) can result in swelling of the hair cell nucleus and of the hair cell itself, as well as an increase in lysosomal granules (which store self-destructing enzymes), distortion of the cuticular plate, and a variety of changes to the stereocilia. If the intracellular breakdown is mild, the cell may be capable of recovery. If the damage is severe, cell death (apoptosis) is initiated and the hair cell will degenerate completely. A short time after acoustic trauma, it is unusual to observe hair cells that are partially damaged; the cells appear to either recover or degenerate completely.

## 5. Damage to Stereocilia

**Figure 1:**
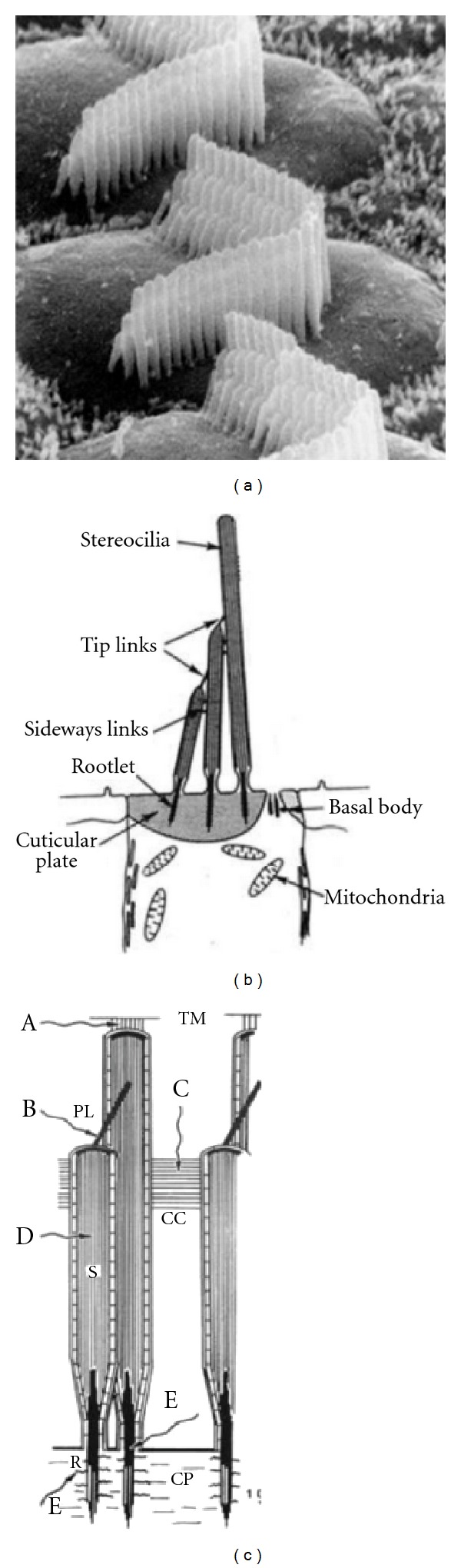
Structural arrangement of hair cell stereocilia. (a) Scanning electron micrograph normal outer hair cells. ((b) and (c)) Structure and linkages of stereocilia. In the right-hand panel, adapted from Saunders et al. [1], potential sites for damage due to acoustic trauma are indicated (A–E; see text).

The stereocilia are key elements in the mechanoelectrical transduction process, and the effects of noise trauma on these structures have been extensively studied. Scanning electron microscopy has proved a particularly useful tool (e.g., [7–13]).  Figure 1 illustrates the structures of the stereocilia that are vulnerable to damage. The left-hand scanning electron micrograph shows normal outer hair cell stereocilia that are stiff, arranged in a stacked “organ-pipe” arrangement. They are held together by cross-linkages at their tips and along their length, and, for the outer hair cells, the longest stereocilia are embedded firmly into the overlying tectorial membrane (e.g., [1, 14–16]). The right-hand panel (based on a review by Saunders et al. [1]) indicates the structures vulnerable to mechanical damage: (A) connection of (longest) stereocilia with the tectorial membrane; (B) tip-links; (C) crosslinks between stereocilia, actin filaments, and other structural proteins; (E) rootlet of stereocilia in hair cell body.

**Figure 2:**
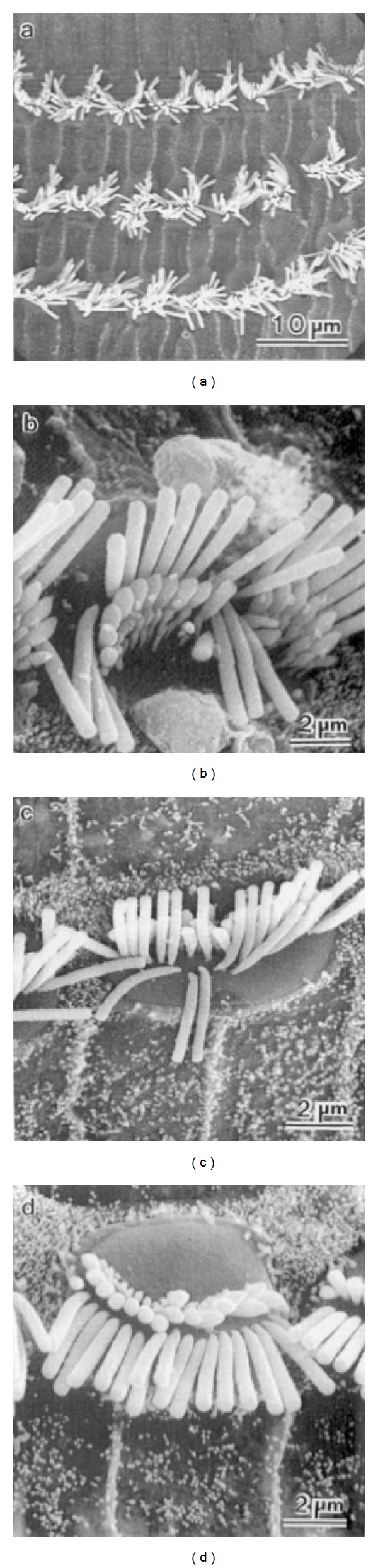
Some early effects of cochlear acoustic overstimulation on cochlear structure, disarranged and floppy hair cell stereocilia.

Examples of stereociliar damage caused by acoustic trauma are shown in Figure 2. Floppy, collapsed, or disarranged stereocilia are some of the first observable effects of acoustic injury, and these often accompany some of the intracellular changes mentioned above. The cross-linkages that normally hold the stereocilia together are often broken. Attachments of the outer hair cell stereocilia to the tectorial membrane may break. Floppy stereocilia appear to have lost their rigidity. The actin protein microfilaments that make up the stereocilium [17, 18] normally provide a very rigid structure that appears to break down after acoustic trauma. Breaks in the stereociliar rootlet or its attachments into the cuticular plate will cause disarray or collapse [19–26].

**Figure 3:**
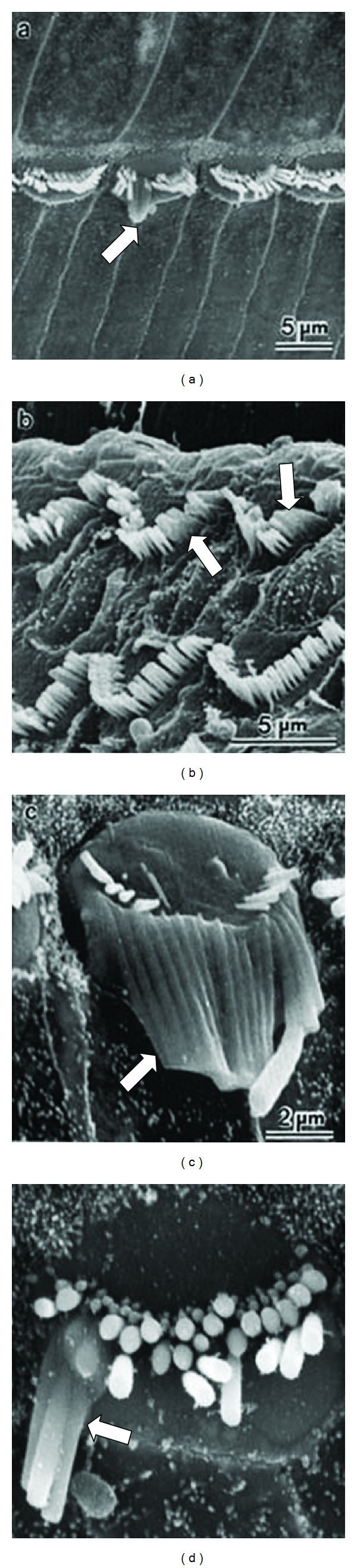
Fused stereocilia observed in the final stages of hair cell degeneration after acoustic trauma.

**Figure 4:**
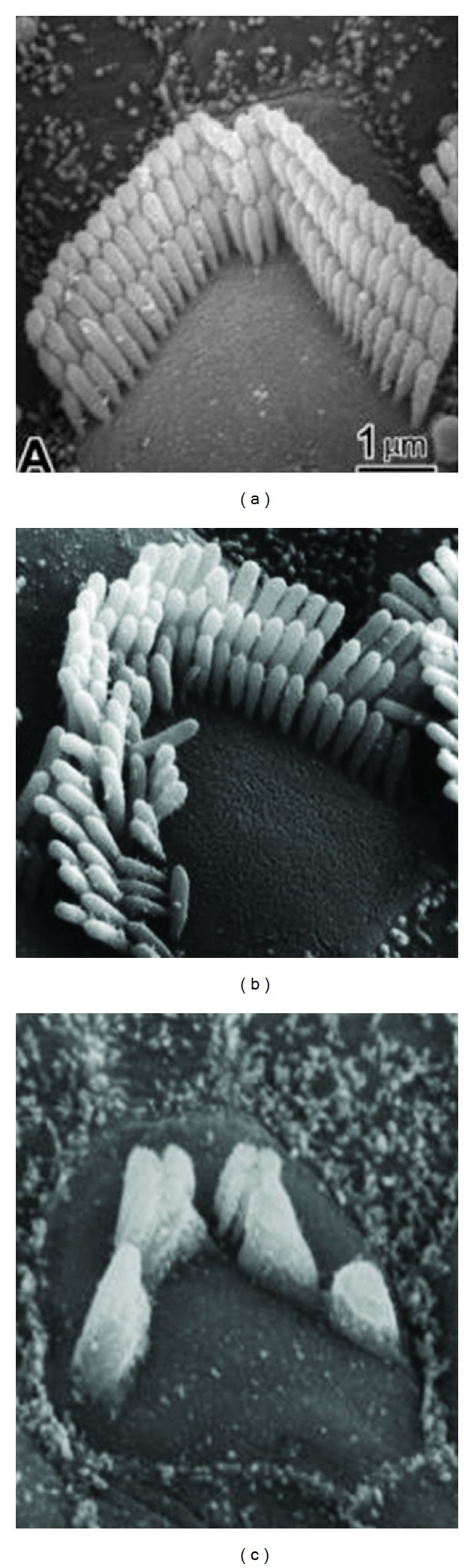
Normal outer hair cell (a) and a damaged and a degenerating outer hair cells (c) after acoustic trauma.

The images of Figure 2 represent the initial effects of noise trauma. Further stages in damage are illustrated in Figure 3, where stereocilia have become fused together so as to share a common surface membrane (see arrows). There can also be fusion of cilia with the apical surface of the cell. It is not clear if this fusion process represents a direct mechanical effect of acoustic stimulation or whether it represents a more general autolytic process; observations of similar fusion after ototoxic drug damage [27] suggest the latter.  Figure 4 shows examples of the sequence from normal (left) to the degenerating hair cell (right).

**Figure 5:**
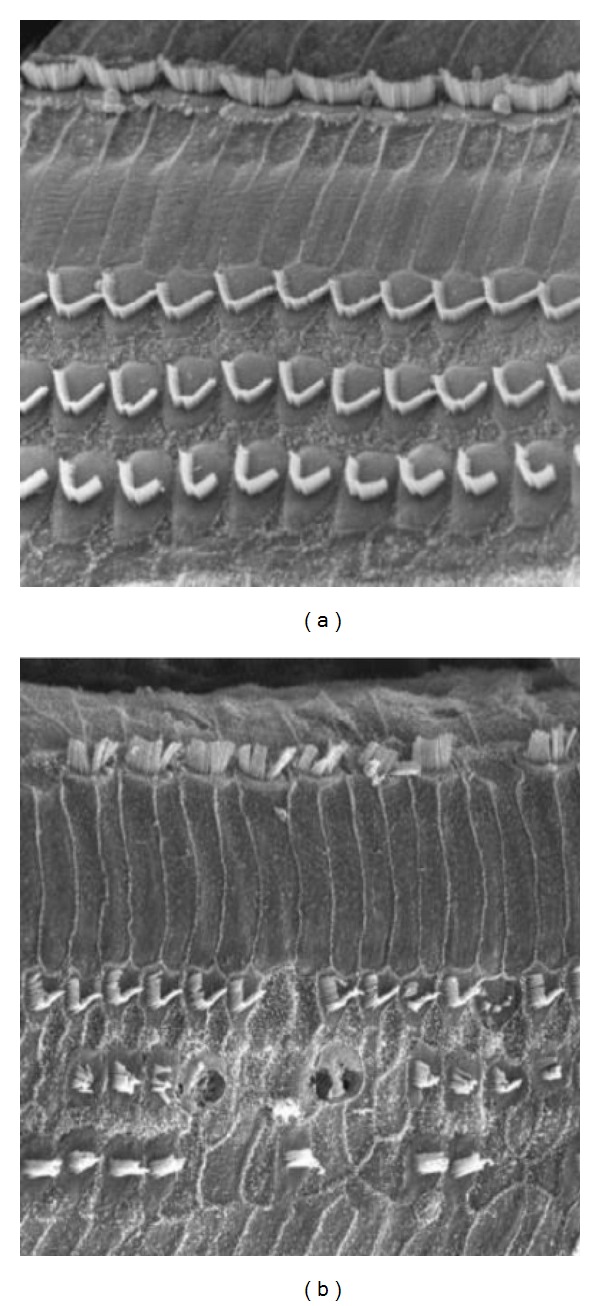
Scanning electron micrographs of the cochlear sensory epithelium before (a) and weeks after recovery from acoustic trauma (b).

Figure 5 represents the cochlear sensory epithelium before and long-term (2 months) postacoustic trauma. There is generally more damage to outer hair cells than inners (e.g., [10, 19, 21]). This could be the result of outer hair cell stereocilia being more firmly attached to the tectorial membrane than those of the inner hair cells and thus more excessively displaced by mechanical vibrations in the cochlea. There are also metabolic theories suggesting that outer hair cells are more susceptible to the metabolic exhaustion effects of acoustic trauma. Note also that while a small degree of disarray of stereocilia can exist after some months, generally cells damaged beyond a certain point (as yet undefined) degenerate completely, leaving the conspicuous gaps in the reticular lamina.

## 6. Permanent versus Reversible Hair Cell Damage

What cochlear pathology is reversible and might therefore underlie temporary threshold shifts? What damage is irreversible and related to permanent threshold shift? There is some evidence that very mild damage to stereocilia can recover [16, 23, 26, 31]. For example, in studies involving the direct observation and measurement of stereociliar bundle deflection, a 10-minute period of overstimulation causes the stiffness of the bundle to decrease. During a 15-minute recovery period, there is a return of stiffness to the stereocilia. In the presence of metabolic inhibitors, this recovery process is impaired, implying that it requires some active metabolic process. These studies indicate that a possible cause of temporary changes in hearing sensitivity could relate to a loss of stereociliar stiffness, but the exact locus of these changes is unknown. It could be in the protein structure of the stereocilium itself or at its region of attachment to the hair cell (cuticular plate).

## 7. Electrophysiological Studies of Acoustic Trauma

Pioneering studies on the effects of noise trauma involved the recording of gross electrophysiological potentials, such as the cochlear microphonics and compound action potentials, to monitor cochlear function (e.g., [2, 32]). The development of more sensitive techniques, in particular microelectrode recording from single neurons, has enabled a more detailed study of the elects of noise damage. In general, one can distinguish two types of experiments. First, there are acute studies in which response properties of cochlear neurons are recorded during exposure of the ear to acoustic trauma (e.g., [21, 28, 33–35]), secondly, chronic studies in which responses of single neurons are recorded from subjects some time after their exposure to intense noise [21, 22, 36, 37]. From such research, we have a more comprehensive picture of the deleterious effects of acoustic trauma on cochlear function. Some of these studies are briefly surveyed here.

**Figure 6:**
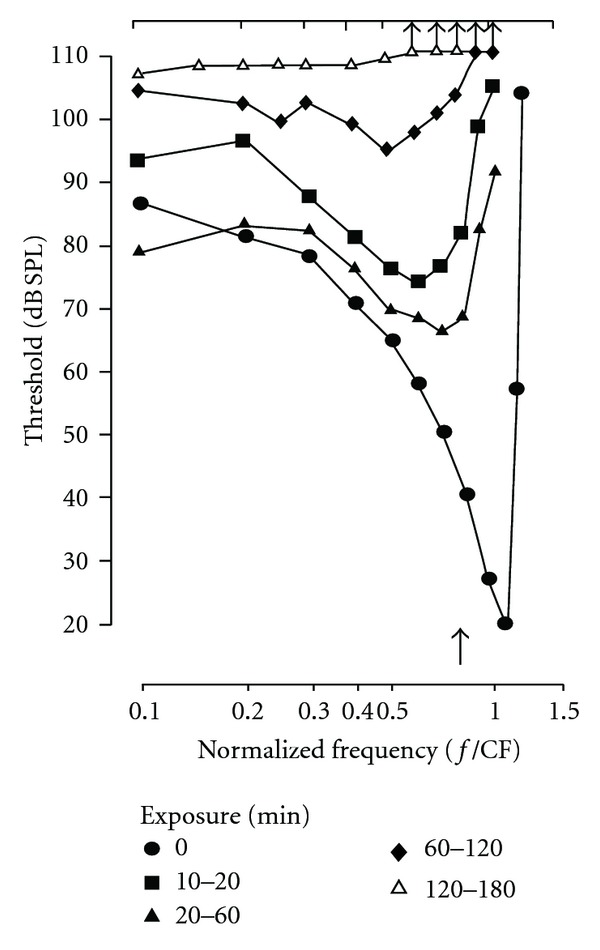
The deterioration in threshold and frequency tuning characteristics of cochlear neurons during loud sound exposure (pulsed tone 16 kHz at 100 dB SPL) for up to 3 hours. Diagram adapted from Cody and Johnstone [28].

Figure 6, adapted from the work of Cody and Johnstone [28], illustrates changes in frequency threshold (tuning) curves of spiral ganglion neurons in a guinea pig recorded before and during three hours of exposure to a 16 kHz pulsed tone at 100 dB SPL. The sequence of change starts with an elevation of threshold of the sharply tuned tip region of tuning curve, the response area; the low frequency “tail” thresholds are not initially changed. For neurons with high characteristic frequency (CF) such as shown in the example of Figure 6, there is a lowering of CF during the exposure and a gradual enlargement of the bandwidth of the response area. With continued noise exposure, thresholds at all frequencies become elevated, and eventually the neuron can become totally unresponsive. Clearly, one of the most vulnerable mechanisms is that responsible for the sharp tuning of the neuron.

Liberman and his colleagues [24–26] have been able to make some very accurate correlations between cochlear neuron response properties and the structural abnormalities of the hair cells after noise trauma. After recording from a cochlear neuron, they filled the cell with horseradish peroxidase, such that it could be traced back to its origin within the organ of Corti. These authors have recorded from neurons during noise exposure to produce a temporary threshold shift. They report that a substantial threshold change up to 60 dB can occur with very little obvious structural change to the cochlear hair cells, at least as assessed with light microscopy. The only clear changes were dendritic swellings of cochlear afferents near to the inner hair cells. Further studies, however, hint that very subtle reversible changes in the disorder of the stereocilia may be responsible for temporary threshold shifts. As discussed previously, this damage might involve a partial breakdown in the connections holding stereocilia together or changes to protein filaments within the stereocilia or their rootlets (e.g., [6, 10, 19]).

**Figure 7:**
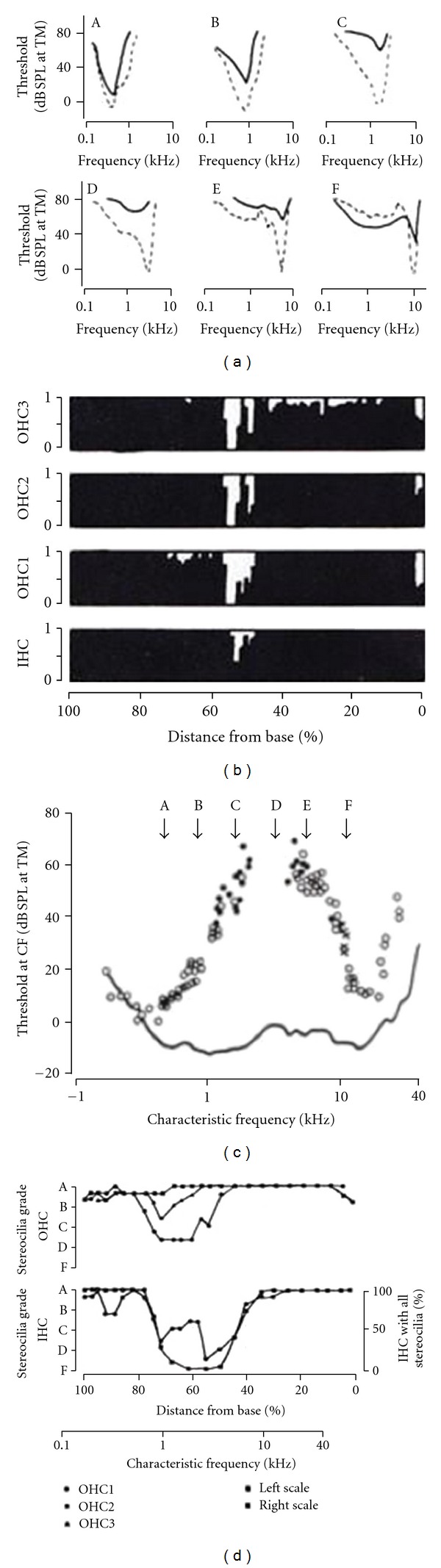
Physiological and anatomical changes in a cochlea with permanent threshold shift resulting from noise exposure. The graphs on the right map the damage to the cochlear hair cells in terms of presence or absence of cells (top panel) and proportion of hair cells with stereociliar abnormality (lower panel). On the top right is a sample of frequency tuning curves of neurons originating in cochlear frequency regions (A–F) near to the damaged cochlear area. The dashed curves represent normal tuning from control animals. The data points in the lower left-hand diagram are the minimum thresholds of cochlear neurons compared with normal values (continuous curve). Adapted from Liberman and Mulroy [21].

Experiments that have investigated the long-term effects of acoustic trauma on cochlear neural responses reveal the chronic functional changes that are likely present in patients with noise induced hearing loss.  Figure 7 shows the results of an elegant study by Liberman and Mulroy [21] in which a cat was exposed to a band of noise centered at 3 kHz at 115 dB SPL for two hours. Two months later recordings were made from a large number of cochlear neurons. The cyto-cochleogram (upper right) indicates the number of outer and inner hair cells remaining after the acoustic trauma. The graphs in the lower right indicate degrees of disorder to the stereocilia on the remaining hair cells. The minimum thresholds of the neuron responses are indicated by the data points in the lower left-hand panel (here the continuous curve represents the thresholds in a normal animal). It is clear that the frequency range over which there are minimum threshold elevations is much wider than that defined by the loss of hair cells along the length of the cochlea and more correlated with areas in which there is some subtle stereociliar damage. The top left-hand panel presents a sample of the tuning curves recorded from neurons originating near to and on both sides of the site of the cochlear lesion. Typical types of abnormal tuning curves (continuous lines) are illustrated and are compared with tuning curves from normal cochleas (dashed curves). Neurons originating close to the lesion (e.g., C, D, and E) show high threshold and a marked deterioration in tuning.

This loss of cochlear frequency selectivity translates into an inability of the auditory system to analyze frequency components in complex sound and is, thus, a contributing factor to poor speech intelligibility in patients with moderate to severe hearing loss from acoustic trauma (e.g., [38–42]).

## 8. Noise Induced Hearing Loss Only Starts at the Cochlea

In addition to the direct trauma that intense sounds can cause to hair cells, there are also secondary effects that can cause further damage. Just as with a brain injury (e.g., stroke), there can be initial restricted lesion, but then cellular byproducts that are released, for example, oxidative free radicals or excessive amounts of neurotransmitter, can cause more extensive further damage. It is also possible that prolonged acoustic overstimulation can lead to local vascular damage and a cochlear hypoxia that in turn can cause damage to hair cells [43].

One issue that has been of some interest to clinicians is the question of whether medical treatment with steroids (given either systemically or locally by transtympanic injection) has any utility in treating acute sensorineural hearing loss, including noise induced sudden deafness. The concept behind such treatment is that the progression of damage that comes subsequent to the initial hair cell damage (as outlined above) may be prevented. This issue has been controversial for some decades, and very recent discussion continues with an editorial by Piccirillo in JAMA [44]. Commenting on studies comparing oral versus transtympanic steroid administration, he concludes that the recent work “does not answer the lingering question of whether there is any benefit of steroids for the patient with sudden sensorineural hearing loss.” This conclusion is similar to that from a systematic review [45], a meta-analysis [46], and a Cochrane review [47]. Theoretically, it is possible that steroid treatment immediately (within minutes) after cochlear trauma could achieve some reduction in secondary damage, but this is rarely the situation clinically.

## 9. Temporary Threshold Shift and Tinnitus

Other than the loss of hearing sensitivity, there are two other common symptoms related to noise induced hearing loss. One is temporary threshold shift; the other is tinnitus or ringing in the ears. Regarding the former, after exposure to a period of loud sound, there can be a “temporary” mild hearing loss. We have all experienced this after a long air flight or bus journey or following a loud music concert. We tend not to worry about such experiences, in part because there appears to be a full recovery. However, it is widely supposed that repeated episodes can result in permanent changes. As we reviewed above, it is likely that the noise exposure resulting in temporary threshold shift alters the delicate micromechanics of the cochlea or the structure of stereocilia and their delicate linkages (see Figure 1). For both ourselves and patients alike, it is wise to avoid (if possible) any noise exposure that results in temporary hearing threshold changes.

There are a number of types of tinnitus, and not all result from cochlear damage. Often, tinnitus is transient, and indeed it is normal to occasionally hear a brief ringing in the ear that dies away within a few seconds. However, when chronic tinnitus is experienced after exposure to loud sounds, it is not just a warning sign but a clear manifestation of cochlear injury. Consider the ringing sound to be caused by hair cells and neurons actually in the process of dying. Such cells generate a neural injury discharge because the cell membrane breakdown causes repeated depolarization (excitation) and/or uncontrolled release of neurotransmitter. In the case of a severe acoustic trauma, tinnitus can persist and become permanent. It has been suggested that the initial neural injury discharge sets up and/or strengthens synaptic connections in a network of auditory neurons at a more central (e.g., cortical) brain level and that these cells continue to fire spontaneously. This could be because a local positive feedback circuit is established or because local inhibitory neuron activity is reduced [48]. Chronic tinnitus can be as devastating on quality of life as a hearing loss. Clearly any recreational activity that induces tinnitus should be avoided.

## 10. Noise Induced Hearing Loss Is a Growing Problem

We are all born with a fixed number of cochlear hair cells. In humans (and all other mammals), they do not regenerate, and so we should take preventative care of these cells. In some vertebrates, for example, in birds, hair cells do regenerate after damage. Actually new hair cells develop from local supporting cells that act like a type of stem cell. In our vestibular sense organs, the hair cells are capable of regeneration, but in the cochlea this does not happen. There is presently a considerable research effort to determine if cochlear hair cells can be made to regenerate, either by providing suitable growth hormones or by finding a genetic switch to turn on the cell differentiation process (e.g., [49–51]). Presently, however, the fact remains that if we kill hair cells by noise exposure, they are lost forever.

Here is a survey of some of the current literature on the noise induced hearing loss in children and young adults. This is not an exhaustive or systematic review, but a representative sample of studies which all point to the growing problem. It has to be recognized that in this area, there is no definitive, “level A” evidence, that is, prospective, randomized controlled studies. However, there are numerous other study types, most of which are cautionary.

In terms of general population studies, a report from a large-scale US national health survey indicates that 12–15% of school-age children have some hearing deficits attributable to noise exposure [52]. In Canada there are no large surveys that specifically address noise induced hearing loss; however, Statistics Canada data [53] indicate that 13% of children (up to 14 years) have some hearing disability but does not separate out specific etiology. In a large US survey [54] of audiograms of 2526 young people entering an industrial work force, 16% had a significant high frequency hearing loss and 20% had audiometric pattern (4 kHz notch) consistent with noise trauma. In a similar type of survey by the Workers Compensation Board of British Columbia, Canada [55], over 20% had some early signs of hearing loss (but the study did not specifically separate out noise induced loss).

There are many smaller scale studies addressing noise induced hearing loss in children. In a Scandinavian research [56], hearing tests in 538 teenage boys revealed a hearing loss greater than 15 dB in 15% and that the characteristics of the loss (notch in the audiogram) indicated that the majority were related to noise exposure. Similarly, a German review of clinical data [57] estimates that one in ten adolescents has some degree of noise induced hearing loss from “leisure time noise.” In a recent Chinese study of 120 young users of “personal listening devices,” impaired hearing (>25 dB loss) was found in 14% of ears [58]. A French audiometric survey of 1364 young subjects found evidence of hearing problems in 12% of the general population, and in a sub-group that often attended rock concerts or used “personal cassette players” for more than 7 hrs a week, 66% had a hearing loss [59]. A similar finding was reported in a smaller group (*N* = 24) of German teenagers [60].

A number of studies have specifically focused on possible hearing loss from personal music players. In an interview study of 490 Korean adolescents, a strong conclusion was that long-term exposure to music player can have “a deleterious effect on hearing thresholds.” The authors ascribe the hearing losses to the use of personal entertainment devices and attendance of concerts where amplified sounds are enough to cause noise induced hearing loss [61]. Recognizing that there is a real problem, many studies have focused on some of the specific causes, such as very loud signals from some cordless telephones [62], the types of headphones or earphones used in personal entertainment devices [63], and the actual levels of sound that are generated by earphone transducers [64, 65]. In addition, there are numerous other reports on other possible sources of noise trauma for children, including very noisy toys, cap guns, and fireworks. Other research has assessed the risks of noise induced hearing loss at specific entertainment venues such as rock concerts [66] and “urban music clubs” [67]. There is even a published report from a Canadian research team with the title “Can hockey playoffs harm your hearing?” [68]. All of these reports and studies suggest that there is a potential problem with noise induced hearing loss at certain entertainment events. In noise induced hearing loss from very high-level sound exposures, tinnitus is often reported. For example, in a Swedish study of 55 boys (ages 8–20) who were seeking help for tinnitus, the majority were found to have been exposed to excessive noise, mostly from recreational music [69]. One study suggested that after short-term exposure to (over)amplified music, tinnitus might be more of a problem than any hearing deficit [60].

To balance the evidence, some studies have concluded from their data that there is no clear link between recreational noise exposure and hearing loss. For example, one research group concluded that most young users of personal listening devices are at low risk for noise induced hearing loss [70]. However, these authors cautiously admit that their study group did not include certain high-risk populations with greater noise exposures and go on to strongly recommend educational sessions about the dangers of noise exposure. An extensive Australian survey [71] also concluded that there was “no widespread hearing loss caused by recreational noise,” but does warn that “if recreational patterns remain the same,” teenagers will be at high risk for noise induced hearing loss by their mid-twenties. To summarize on a cautious note, a recent general review of the issue of noise induced hearing loss in relation to school-aged children [72] concludes that it is a major cause of hearing loss (in the US) and that hearing impairment among children and teenagers is on the increase due mostly to “voluntary exposure” to loud noise (i.e., using personal entertainment devices or attending amplified sound concerts).

## 11. Noise Induced Hearing Loss May Be Manifested Later in Life

There is a strongly held view (which this author also holds) that noise exposure effects are cumulative. Thus, in the short term, the effects of noise overstimulation may not be obvious, but the accumulated effects of damaging episodes eventually lead to significant hearing deficits. An important point here concerns the redundancy of hair cells in the cochlea. There are many more sensory elements than we need, and so considerable cell loss can occur before there are clinical signs of a problem. However, with repeated insults, our fixed complement of hair cells eventually runs out. This is one reason why noise induced damage in early years may not be immediately manifested but may become a problem in later life.

## 12. What Are the Full Consequences of Hearing Loss in Children?

For most healthcare professionals, hearing loss is largely described by the results of clinical tests such as the audiogram or speech threshold measures. In infants, hearing thresholds can be assessed objectively using auditory evoked potentials or otoacoustic emissions.

Typically a deficit will be described as a hearing threshold loss in decibels (dB). It is important to realize that such basic threshold loss measures do not fully reflect a hearing “problem.” It is common to have a child with relatively good hearing thresholds but with significant problems in understanding speech. The ability to understand complex sounds can be reduced before pure tone audiometric threshold shifts are clinically significant. In the above review of the cochlear changes that accompany noise induced hearing loss, it was noted that deterioration in cochlear frequency analysis always accompanies the threshold elevation and is an important part of the overall deterioration in hearing function. In assessing possible noise induced hearing loss in children, clinicians might want to carry out or recommend a comprehensive hearing evaluation including speech discrimination tests.

Beyond clinical tests results, there are broader ways of looking at hearing disability. The World Health Organization (WHO) has a scheme for assessing and describing hearing problems. This model distinguishes (1) impairment, (2) disability, and (3) handicap. Impairment is the actual loss of sensory function such as quantified by the clinical tests mentioned above. Disability is the “activity limitation” of an individual that results from the impairment (e.g., a child might not understand what you say and needs to ask you to repeat words). Handicap is a measure of “participation restriction,” that is, activities that a child may not be able to do because of the hearing problem. This might include making friends, keeping up at school, or being excluded from training for a certain career. For a child with noise induced hearing loss, the degree of deficit is likely to be mild or moderate, as opposed to severe or profound. However, such a loss might still be a barrier to effective communication, especially in noisy environments, such as the school classroom. It should also be recognized that mild to moderate hearing losses may not be immediately apparent to a child (in the same way that many older persons do not recognize that they have age-related hearing loss). Parents and clinicians should be vigilant and remember that measures of speech discrimination may more accurately reveal a hearing problem than simple (threshold) audiogram or hearing screening test.

In a very young child, hearing problems can delay language development, and certainly if information is being missed at school, educational achievements can be reduced. For adolescents, communication difficulties can lead to social isolation, and there have been reports of suicide resulting from such situations. If hearing aid use is warranted, the adolescent may also have problems with the cosmetic appearance of the device or the stigma attached. The child may decide not to use the aid or choose to retreat to a small group or social isolation. In any case, it can be assumed that there will be quality of life implications. The quality of life impact may also be felt at a later age when job opportunities are restricted because of the hearing problem itself or a reduced educational attainment. The impact may well also be an economic one.

## 13. Practical Advice about Noise Induced Hearing Loss

The noise in our environment is no longer all “natural” and there numerous sources of amplified sounds that can damage hearing. In (western-based) industry, business, and the military, there is legislation or strict safety guidelines relating to noise exposure. In the areas of public entertainment (discos, rock concerts, sports stadiums) and personal entertainment devices, (MP3 or CD players and electronic games), regulations are not fully in place, and even if they were, it would be difficult to achieve compliance.

Fortunately, there are numerous public awareness campaigns on the dangers of noise exposure, and there are some educational programs in schools that teach children that hearing loss can result from listening to loud sounds (by analogy, just as many of us were told at school not to look directly at the sun!). Much useful information is now available online. A very rich and informative website is WISE EARS [73] launched in 1999 by the US National Institute on Deafness and other Communication Disorders (NIDCD) together with the National Institute for Occupational Safety and Health (NIOSH). This provides a wealth of useful information for children, teachers, parents, and the public at large. Also sponsored by the National Institutes of Health (NIH, including NIDCD) and the US Government is IT'S A NOISY PLANET [74]. This is targeted to children in the 8–12 year age range, as well as parents and teachers.

**Figure 8:**
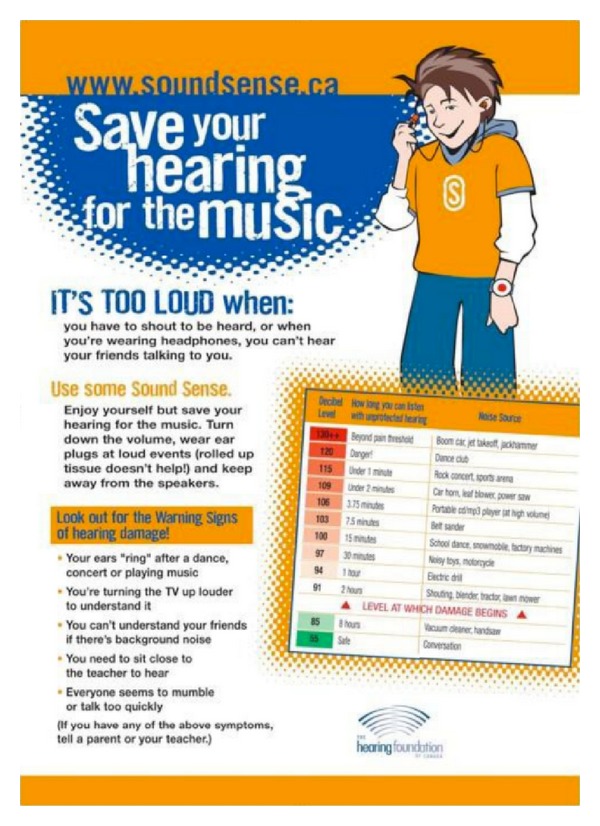
Sound Sense poster from the Hearing Foundation of Canada [29, 30]. This is a part of an educational campaign for school children about the prevention of noise induced hearing loss using the slogan “save your hearing for the music.”

There are numerous websites offering advice and promoting awareness. For example, KEEP IT HEAR [75] is “A Noise Induced Hearing Loss Awareness Campaign.” For school-age children there is LISTEN TO YOUR BUDS, “Keeping Kids Safe in Sound” [76]. HEAR-IT (YOUTH) [77] is a general web resource about hearing loss problems with a special section targeted at young people about noise induced hearing loss. Another useful instructional website is named DANGEROUS DECIBELS. This is a joint project between the Oregon Museum of Science and Industry and the Oregon Hearing Research Center [78]. The Hearing Foundation of Canada [29] has introduced a successful program called “Sound Sense” [30] into many schools, where age-appropriate materials provide children with the facts and encourage prevention. This foundation and others also attempt to get the prevention message out via web-based information portals and advertisements.  Figure 8 is an informative poster distributed by The Hearing Foundation of Canada and provides a guide to the levels of sounds that are a potential risk. This “Sound Sense” poster is targeted toward school-age children.

Note on the table of various levels of example sounds-there is also an indication of how long an exposure to that sound can be considered safe. This is an important concept with regard to noise induced hearing loss. It is not just the sound intensity but also the duration of the exposure that determines its potential to cause cochlear damage. The efficacy of this program in changing behavior has been proven effective in a validation study [79].

In recent years, the manufacturers and distributors of personal entertainment devices have been providing (in package) warnings and practical advice relating to the risks of noise induced hearing loss. It would be good to suppose that this was good corporate responsibility, as opposed to just being a hedge against possible litigation. In any case, such instructions, if attended to, can help prevent noise induced hearing loss.

## 14. Some Advice to Parents and Children

Many parents (and children) have concerns about the levels of sound exposure from personal entertainment devices and the risks of hearing loss. One common problem is a tendency to turn up the device volume to overcome the surrounding noise, which itself can be substantial. It is useful to suggest that adjustment of the device volume is made in a relatively quiet environment, to a level that is comfortable and then avoid increasing the volume further even in noisy settings. Our general auditory experience can tell us what is comfortable and what is too loud; perhaps parents should help younger children find that level. Some other useful advice is to get a child into the habit of checking if others nearby can also hear the music. If so it may be set too loud, although this of course will depend on the type of earphone in use. Mention was made above of the notion that it is not just the intensity of noise that is a problem, but also the duration of exposure. In this regard, for a young person who is constantly listening to music, it is advisable to take periodic 15–20 minute breaks, to allow the inner ear to “recover.”

Another issue relates to the type of earphone or headphone used. The least risky in terms of the potential to do damage are loose fit earbuds that do not insert tightly into the ear canal. They are typically small transducers and do not output acoustic energy directly into the confined space of the ear canal. On the other hand, the listener is not insulated from the environmental noise and thus there is often a tendency to increase volume accordingly. Perhaps for the “careless” child, this type of earphone is the best. If the child is more responsible, then a type of earphone that blocks the outside noise can be recommended. This can provide the ear with a better sound (improved signal to noise ratio) and obviate the need to increase volume to compete with environmental noise. These can be earbuds that fit snugly right into the ear canal or a larger headphone that fits against or around the ear. The downside for these transducers is that they can actually produce very intense signals either because sound energy is transmitted into a closed space or because of the size of the transducer diaphragm in the case of large headphones. For the serious music lover, active noise- reduction earphones are a nice luxury, but they are not very practical for children and may isolate an individual too much from the outside world.

## 15. In Summary

This paper started by describing the cochlear hair cell damage that can result from acoustic trauma and has emphasized the fact that there is little recovery and no regeneration of damaged hair cells. In this sense, there is really no treatment for noise induced hearing loss other than hearing aids that cannot fully restore normal hearing. With this being the case, all healthcare professionals should pay considerable attention to the education of parents and children about hearing loss prevention.
